# Clinicopathological features and prognosis of intestinal hepatoid adenocarcinoma: evaluation of a pooled case series

**DOI:** 10.18632/oncotarget.23595

**Published:** 2017-12-21

**Authors:** Xiangyu Zeng, Peng Zhang, Hua Xiao, Xiuli Wu, Weizhen Liu, Jun He, Jinbo Gao, Guobin Wang, Xiaoming Shuai, Kaixiong Tao

**Affiliations:** ^1^ Department of Gastrointestinal Surgery, Union Hospital, Tongji Medical College, Huazhong University of Science and Technology, Wuhan 430022, China; ^2^ Department of Gastroduodenal and Pancreatic Surgery, Hunan Cancer Hospital and The Affiliated Cancer Hospital of Xiangya School of Medicine, Central South University, Changsha 410013, China; ^3^ Department of Pathology, Union Hospital, Tongji Medical College, Huazhong University of Science and Technology, Wuhan 430022, China

**Keywords:** hepatoid adenocarcinoma, intestines, stomach, feature, prognosis

## Abstract

**Backgroud:**

Intestinal hepatoid adenocarcinoma (IHA) is a very rare and unique intestinal malignancy. Due to the lack of case series specifically pertaining to IHA, the clinicopathological features and prognosis of it remain unclear.

**Results:**

Of the 42 patients enrolled in this study, 30 (71.4%) were male. Twenty-one cases (50.0%) were located in the colon. Eight cases (19.0%) had accompanying inflammatory bowel disease (IBD). Elevated serum alpha-fetoprotein (AFP) was detected for most patients (25/33, 84.8%). Twenty-five (59.5%) patients received complete resections. Vascular invasion (22/36, 61.1%), lymph node metastasis (28/36, 77.8%) and distant metastasis (21/42, 50.0%) were common. The 1-year progression-free survival (PFS) and disease-specific survival (DSS) of IHA were 26.9% and 30.6%, respectively. Multivariate analysis showed that only pTNM stage was an independent risk factor for PFS and DSS. PFS and DSS in patients with IHA were significantly lower than those with colorectal adenocarcinoma (CA) and hepatoid adenocarcinoma of the stomach (HAS).

**Conclusions:**

IHA most commonly occurred in the colon and accompanied by IBD in several cases. pTNM stage was an independent factor for prognosis. The prognosis of IHA was significantly worse than that of CA and HAS.

**Patients and Methods:**

Clinical data of IHA from four patients managed at our institution between January 2010 and December 2016, and 38 cases from research databases prior to 2017 were retrospectively studied.

## INTRODUCTION

Hepatoid adenocarcinoma (HAC) is a rare and special type of extrahepatic malignancy with an estimated annual incidence of 0.58–0.83 cases per million people [1, 2]. It is distinguished by having foci with characteristics of both hepatocellular differentiation and adenomatous differentiation [3]. HAC is more likely to occur in gastrointestinal (GI) tract organs such as the stomach, esophagus, biliary tract, and pancreas, among which the stomach is the most commonly identified site, due to the fact that the gastric system and liver are derived from the same part of the embryo [4, 5]. Nevertheless, sporadic cases of HAC originating from the intestines have been reported in the literature [6–8].

Intestinal hepatoid adenocarcinoma (IHA) is a scarce and unique malignant tumor in intestines. Clinically, IHA commonly exhibits an elevated serum alpha-fetoprotein (AFP) levels, and usually presents extensive vascular invasion, frequent liver metastasis and oftentimes advanced pTNM stage, all of which may contribute to its extremely poor prognosis [9]. However, extensive data regarding the clinicopathological profiles and prognosis of IHA remain limited, with all studies in the literature being case reports. In this study, we collected the data of 42 patients with IHA, aiming to explore the clinicopathological features and clinical outcomes of IHA. We then investigated the potential factors that may predict prognosis.

## RESULTS

Four patients with IHA (2 jejunoileum, 1 colon and 1 rectum) were admitted to Union Hospital, Tongji Medical College, Huazhong University of Science and Technology between January 2010 and December 2016, with an additional 33 case reports, including 38 patients [6–8, 10–39]. Overall, a total of 42 cases of IHA were identified. The clinicopathological characteristics of the 42 patients with IHA are summarized in Table 1. There were 30 male (71.4%) and 12 female (28.6%) patients, (male to female ratio = 2.5:1). The median age was 56 years (21–75). The most common initial presentation was hematochezia (20/42, 47.6%), followed by abdominal pain (14/42, 33.3%) and others including loss weight, obstruction, weakness or abdominal mass (8/42, 19.1%). Twenty-one cases (50.0%) occurred in the colon, 10 cases (23.8%) in the rectum, 6 cases (14.3%) in the jejunoileum and 5 cases (11.9%) in the duodenum. Eight cases (19.0%) were accompanied by inflammatory bowel disease (IBD).

**Table 1:** Clinicopathological characteristics of 42 patients of IHA

Characteristics	*N* (%)
Age (∑ = 42), median = 56 (21–75)	
≤60	26 (61.9)
>60	16 (38.1)
Sex (∑ = 42)	
Male	30 (71.4)
Female	12 (28.6)
Initial presentation (∑= 42)	
Hematochezia	20 (47.6)
Abdominal pain	14 (33.3)
Others	
Loss weight, Obstruction, Weakness, Abdominal mass	8 (19.1)
Location (∑ = 42)	
Duodenum	5 (11.9)
Jejunoileum	6 (14.3)
Colon	21 (50.0)
Rectum	10 (23.8)
Accompanying IBD	8 (19.0)
Elevated of serum biomarkers (∑ = 33)	
AFP median = 4896 (3 ng/ml–400000 ng/ml)	25 (84.8)
CEA	9 (27.3)
CA19-9	4 (12.1)
Biopsy (∑ = 28)	
IHA	4 (14.3)
Moderate differentiated adenocarcinoma	16 (57.1)
Poorly differentiated adenocarcinoma	8 (28.6)
Tumor size (∑ = 35), median = 6 (1–18)	
<6 cm	17 (48.6)
≥6 cm	18 (51.4)
Portal vein thrombosis (∑ = 38)	4 (10.5)
Distant metastasis (∑ = 21)	
Liver	18 (85.7)
Lung	3 (14.3)
Surgical resection (∑ = 42)	
Complete resection	25 (59.5)
Incomplete resection	15 (35.7)
No surgery	2 (4.8)
Diffentiation (∑ = 36)	
Poorly differentiation	18 (50.0)
Mediated differentiation	18 (50.0)
Depth (∑ = 36)	
T1–T2	8 (22.2)
III	12 (30.0)
IV	21 (52.5)
Immunohistochemistry	
AFP for positive (∑ = 42)	38 (90.5)
CEA for positive (∑ = 34)	30 (88.2)
CK19 positive (∑ = 15)	13 (86.7)
CK20 positive (∑ = 12)	2 (16.7)
CK7 positive (∑ = 12)	2 (16.7)
Neo-/adjuvant therapy (∑ = 42)	4 (9.5)/18 (42.9)

IHA: intestinal hepatoid adenocarcinoma; IBD: inflammatory bowel disease; AFP: alpha-fetoprotein; CEA: carcinoembryonic antigen; CA: carbohydrate antigen.

The majority of patients (25/33, 84.8%) had elevated serum AFP levels ranging from 25 ng/mL to 400000 ng/mL (median, 4896 ng/mL); 9 cases (9/33, 27.3%) had elevated serum carcinoembryonic antigen (CEA) and 4 cases (4/33, 12.1%) had elevated serum carbohydrate antigen (CA)19-9. Four cases (4/38, 10.5%) presented with portal vein thrombosis upon imaging examination. Twenty-eight patients had biopsy through colonoscopy, with 4 cases (14.3%) being diagnosed as IHA, 16 cases (57.1%) were diagnosed as moderated differentiated adenocarcinoma and the remaining 8 cases (28.6%) were diagnosed as poorly differentiated adenocarcinoma.

The tumor sizes ranged from 1 to 18 cm (median, 6 cm; mean, 7.1 cm). Eighteen cases were poorly differentiated (18/36, 50.0%) and 18 cases displayed mediated differentiation (18/36, 50.0%). The tumor infiltrating depth of T1–T2 and T3–T4 were 22.2% (8/36) and 77.8% (28/36), respectively. Twenty-eight cases (28/36, 77.8%) had lymph node metastasis and 22 cases (22/36, 61.1%) had vascular invasion. Twenty-one cases (21/42, 50.0%) presented distant metastasis including 18 cases (85.7%) in the liver and 3 cases (14.3%) in the lungs. According to the 7th AJCC classification, the pTNM stage of I–II, III and IV accounted for 17.5% (7/40), 30.0% (12/40) and 52.5% (21/40), respectively. Immunohistochemistry (IHC) determined that 38 cases were positive for AFP (38/42, 90.5%), 30 cases were positive for CEA (30/34, 88.2%), 13 cases were positive for CK19 (13/15, 86.7%), 2 cases were positive for CK20 (2/12, 16.7%) and 2 cases were positive for CK7 (2/12, 16.7%).

Twenty-five patients underwent complete surgical resection (25/42, 59.5%), 15 cases underwent palliative resection (15/42, 35.7%) and 2 cases did not receive surgery (2/42, 4.8%). Eighteen cases received adjuvant therapy (18/42, 42.9%) and among these cases, 4 cases received neoadjuvant therapy (4/42, 9.5%).

**Figure 1:**
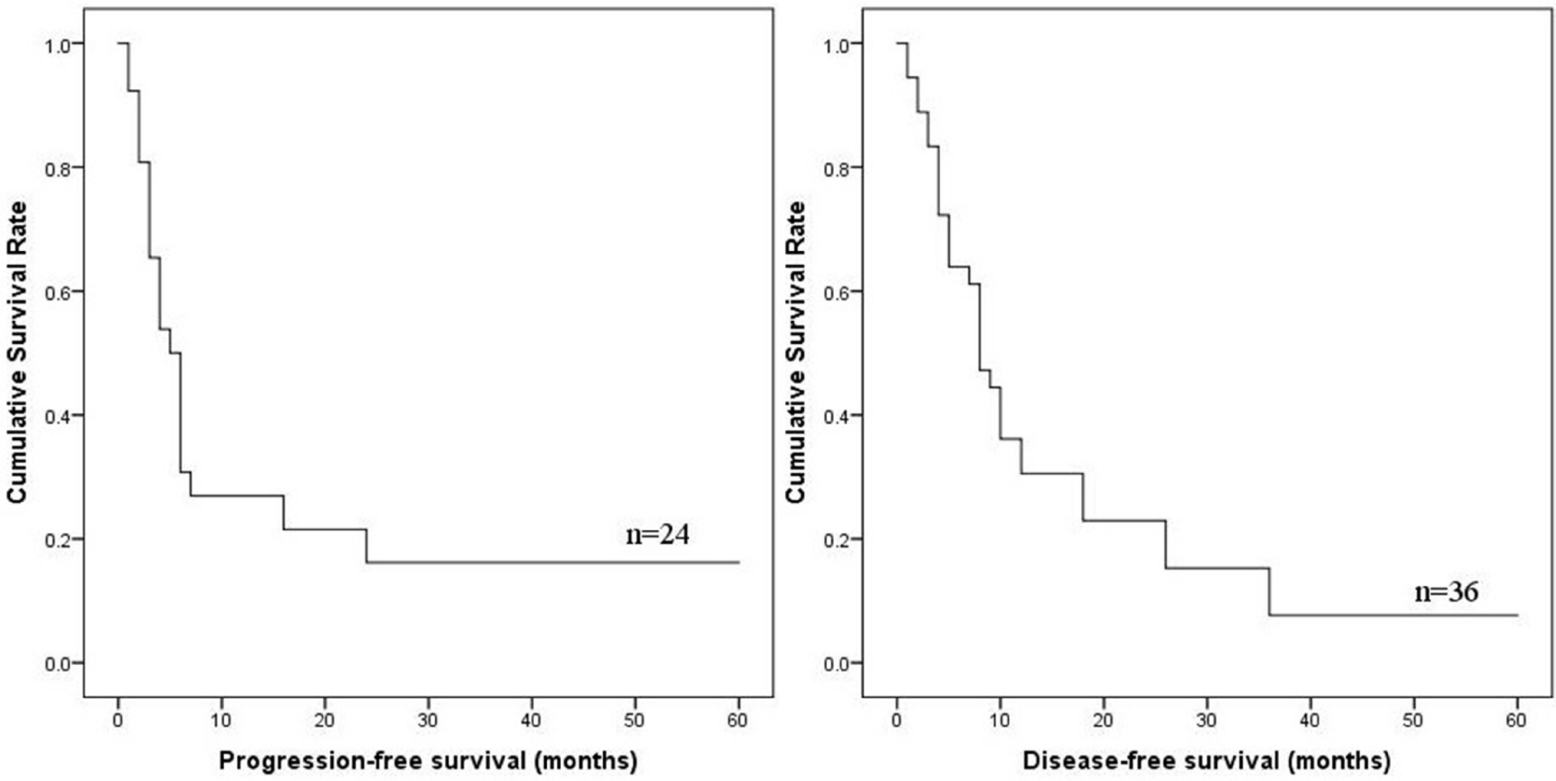
PFS and DSS of IHA

Survival data of the 36 patients were eventually selected for analysis (Table 2). The follow-up period ranged from 1 to 60 months (mean, 11.6 months; median, 8 months). Twenty-one patients underwent disease progression and 29 patients suffered from IHA related death. The 1- and 2-year PFS were 26.9% and 16.2%, respectively. The 1-, 2- and 3-year DSS were 30.6%, 22.9% and 7.6%, respectively. The detailed 1-year DSS data for each pTNM stage were as follows: 66.7% in stage I–II; 22.9% in stage III; 0% in stage IV. The PFS and DSS of IHA are shown in Figure 1.

**Table 2:** Survival data of the 36 cases with IHA

Survival Characteristics	Parameter
Follow-up time (months)	
Mean (m ± SD)	11.6 ±11.5
Median (m, range)	8 (1–60)
Survival data (∑ = 36)	
Progression	21
IHA-related deaths	29
Survival rates (%)	
1-/2-year PFS	26.9/16.2
1-/2-/3-year DSS	30.6/22.9/7.6
1-year DSS of pTNM stage of I–II, III and IV	66.7/50/0

IHA: intestinal hepatoid adenocarcinoma; SD: standard deviation;

PFS: progression-free survival; DSS: disease-specific survival.

**Figure 2:**
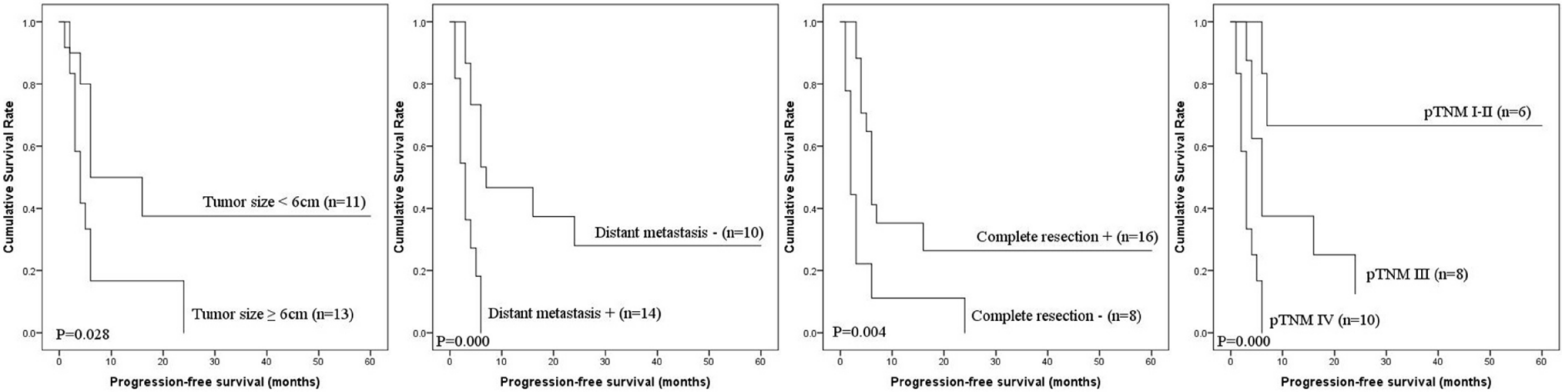
PFS of IHA by univariate analysis

**Figure 3:**
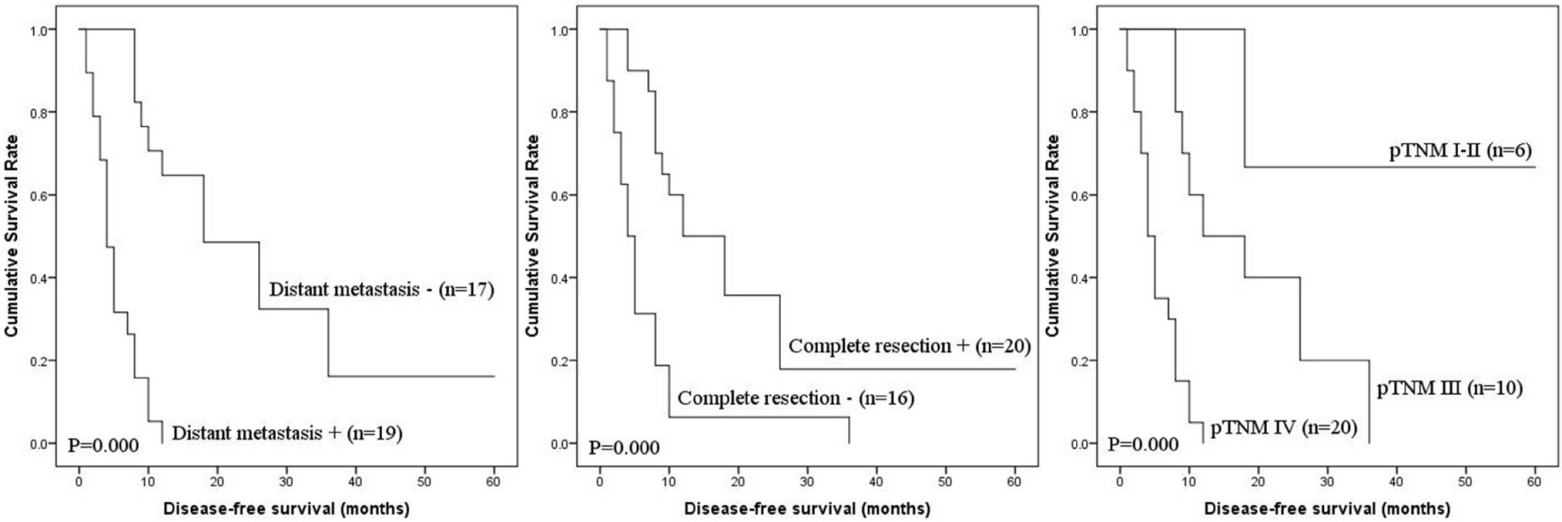
DSS of IHA by univariate analysis

Prognostic factors for PFS and DSS of IHA are shown in Table 3. Univariate analysis showed that tumor size, distant metastasis, complete resection and pTNM stage were prognostic factors for PFS, with distant metastasis, complete resection and pTNM stage as prognostic factors for DSS. Multivariate analysis showed that pTNM stage was an independent risk factor for PFS and DSS. The PFS and DSS of IHA cases according to prognostic factors are shown in Figures 2 and 3.

**Table 3:** Prognostic factors for progression-free survival and disease-specific survival in patients with IHA according to univariate and multivariate analysis

	Univariate Analysis			Multivariate Analysis		
Prognostic Factors	β	Hazard Ratio (95% CI)	*P* Value	β	Hazard Ratio (95% CI)	*P* Value
PFS						
Tumor size	1.015	2.759 (0.996–7.644)	0.028			
Distant metastasis	–1.544	0.214 (0.081–0.565)	0.000			
Complete resection	1.155	3.174 (1.310–7.689)	0.004			
pTNM stage	1.279	3.595 (1.718–7.523)	0.000	1.594	4.923 (1.033–23.468)	0.045
DSS						
Distant metastasis	–2.123	0.120 (0.045–0.315)	0.000			
Complete resection	1.260	3.526 (1.640–7.581)	0.000			
pTNM stage	1.823	0.435 (2.638–14.520)	0.000	1.466	4.331 (1.066–17.598)	0.040

IHA: intestinal hepatoid adenocarcinoma; PFS: progression-free survival; DSS: disease-specific survival; CI: confidence interval.

To compare the clinicopathological features of IHA with hepatoid adenocarcinoma of the stomach (HAS) and CA, we collected patients with HAS from our institution and research databases, while patients with CA were collected from the Surveillance, Epidemiology, and End Results (SEER) statistics program (2004–2014). Ultimately, 328 cases of HAS and 219,256 patients with CA were enrolled (Table 4). The results showed that only age (*P* = 0.012), histologic type (*P* = 0.009), and pTNM stage (*P* = 0.023) were statistically different between IHA and HAS cohorts.

**Table 4:** Comparison of selected clinicopathological parameters between IHA and CA as well as IHA and HAS

Characteristics	IHA	HAS	*P* Value	CA
Age (y)			0.012	
≤60	26	91		65010
>60	16	132		154246
Sex			0.421	
Male	30	235		117820
Female	12	70		101436
Tumor size (cm)			0.911	
≤6 cm	19	77		141682
≥6 cm	18	70		77574
Portal vein thrombosis			0.139	
Present	4	32		_
Absent	34	120		_
Distant metastasis			0.100	
Present	21	121		46913
Absent	21	207		164203
Histologic type			0.009	
Poorly differentiated	18	102		36737
Well/moderately differentiated	18	38		161162
Depth			0.307	
T1–T2	8	64		52953
T3–T4	28	145		143684
Vascular invasion			0.302	
Present	22	199		_
Absent	14	87		_
Lymph node metastasis			0.487	
Present	28	222		85425
Absent	8	61		117816
pTNM stage			0.023	
I–II	7	64		99661
III	12	87		59751
IV	21	63		46913

IHA: intestinal hepatoid adenocarcinoma; CA: colorectal adenocarcinoma; HAS: hepatoid adenocarcinoma of the stomach.

**Figure 4:**
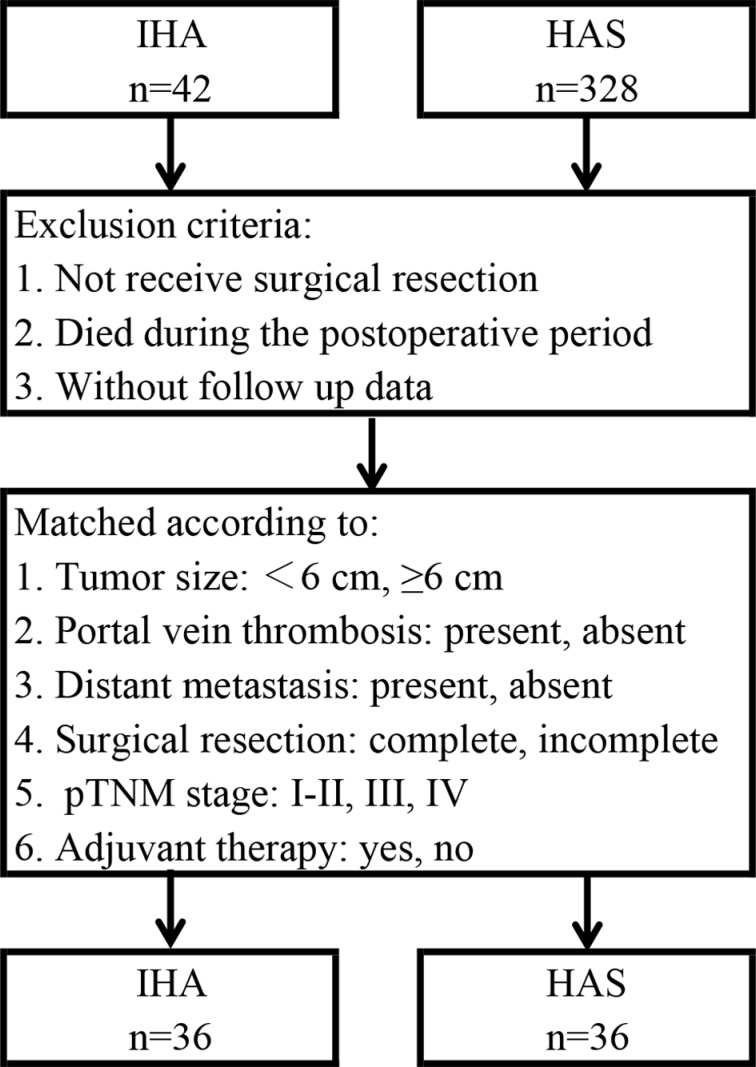
Flow chart of match atrategy between IHA and HAS

**Figure 5:**
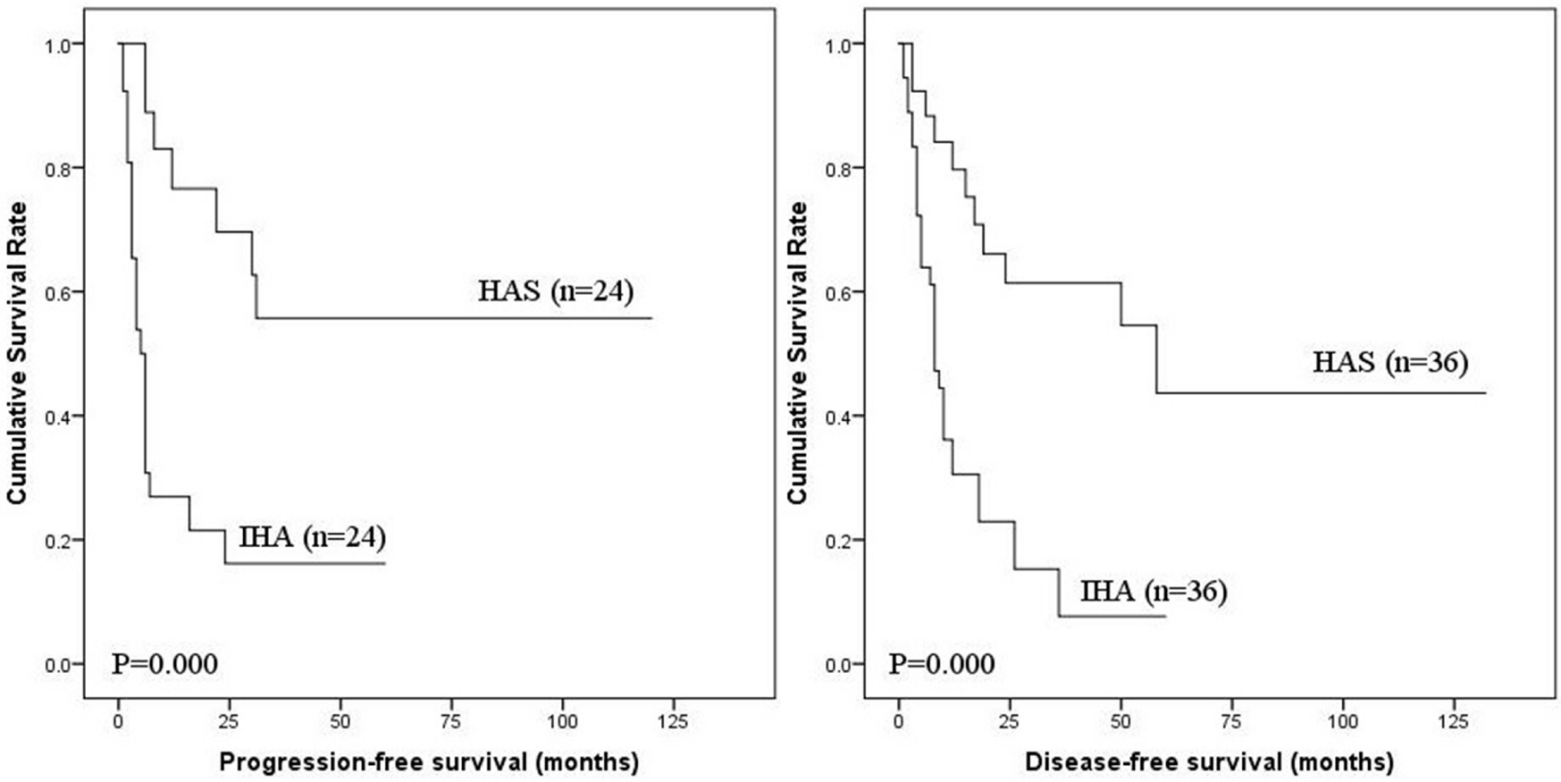
Comparision of PFS and DSS between IHA and HAS

The 1-, 2-, and 3-year DSS of CA calculated from the SEER*Stat program was 84.7%, 76.6% and 70.4%, respectively. The detailed 1-year DSS data for each pTNM stage were 94.5% for stage I–II, 92.8% for stage III, and 59.0% for stage IV. Moreover, to compare the prognosis of IHA and HAS, propensity score matching using a one-to-one scheme without replacement and the nearest-number matching method were conducted according to these prognostic factors described above. The entire process is illustrated in Figure 4. After matching, 36 cases of IHA and 36 cases of HAS were filtered out for analysis (Table 5). There were no intergroup differences in these factors. The PFS and DSS of IHA were significantly lower than that of HAS (both *P* < 0.001) (Figure 5).

**Table 5:** Comparison of predefined variables between IHA and HAS

Characteristics	IHA (*n* = 36)	HAS (*n* = 36)	*P* Value
Age (y)			0.157
≤60	20	14	
>60	16	22	
Sex			0.772
Male	28	29	
Female	8	7	
Tumor size (cm)			0.814
<6 cm	18	19	
≥6 cm	18	17	
Portal vein thrombosis			0.496
Present	4	6	
Absent	32	30	
Distant metastasis			1.000
Present	15	15	
Absent	21	21	
Surgical resection			0.795
Complete resection	25	26	
Incomplete resection	11	10	
pTNM stage			1.000
I–II	7	7	
III	12	12	
IV	17	17	
Adjuvant therapy			1.000
Yes	18	18	
No	18	18	

IHA: intestinal hepatoid adenocarcinoma; HAS: hepatoid adenocarcinoma of the stomach.

## DISCUSSION

In 1970, Bourreilile *et al*. [40] first reported a case of liver metastasis from gastric adenocarcinoma with elevated serum AFP. Fifteen years later, Ishikura *et al*. [41] initially proposed the term “hepatoid adenocarcinoma”, among which this malignancy was most commonly found in the stomach and then followed by the colon. HAS, usually associated with advanced disease stage, liver metastasis, and a poor prognosis, is rare, but well described. Conversely, IHA constitutes a very rare subset of HAC and is typically not well characterized, in regard to its clinicopathological features and prognosis. To the best of our knowledge, this is the first case series describing of IHA. According to the analysis of the 42 cases of IHA from our center and from the literature, we revealed some of the clinicopathological characteristics of IHA, as well as indicated several features significantly associated with this malignancy.

In the present study, we found that IHA commonly occurs around 50 years of age and shows a predilection for the male sex. IHA most frequently arose in the colon, followed by the rectum. The majority of patients presented with hematochezia, especially in the colorectal cases. Moreover, most cases of IHA had increased serum AFP, with extremely high levels (> 1000 ng/mL) typically detected, which is helpful for an objective diagnosis. However, an absence of AFP overproduction did not exclude a diagnosis of IHA and the capacity of tumor tissue to produce AFP had no impact on prognosis. Meanwhile, other serological biomarkers such as CEA and CA 19-9 were always within normal ranges. Interestingly, in our study, 8 patients with IHA (4 in the rectum, 2 in the colon and 2 in the jejunoileum) were accompanied by a long-standing history of IBD. Several studies have reported that IBD may be a specific risk factor for developing malignancy of the intestines, although the exact pathogenesis remains unknown [42, 43]. In addition, it has been hypothesized that chronic inflammation contributes to this appearance, which is similar with other gastrointestinal cancers, such as esophageal cancer originating from Barrett's esophagus, gastric adenocarcinoma due to Helicobacter pylori-associated chronic gastritis, and hepatocellular carcinoma caused by hepatitis B and C viral infections [44]. Therefore, this novel finding strongly suggests that IBD may not only promote to traditional adenocarcinomas, but also to IHA.

An early diagnosis and treatment are crucial to improve the survival of patients with IHA. Unfortunately, a preoperative diagnosis of IHA is exceedingly difficult. First, elevated serum AFP is not a universal marker although it increases in most cases [23, 24]. Moreover, the imaging appearance of IHA is not specific. They usually present as large masses with the same density of the normal liver on baseline imaging and are moderately enhanced after administration of an intravenous iodinated medium, which show no differences with other malignant tumors [30, 32]. Additionally, even preoperative biopsy can rarely obtain a definitive diagnosis of IHA. There were only 4 patients (14.3%) diagnosed as IHA with biopsy through colonoscopy in our study. A definitive diagnosis of IHA depends on the histological morphology of both aberrant hepatocellular differentiation and adenocarcinomatous differentiation, in addition to immunohistochemical features. HepPar1 is always found to be positive, owing to its high sensitivity and specificity for hepatocyte differentiation [33]. Moreover, IHA displays moderate to strong positive reactivity for both AFP and CEA with positive cytoplasmic staining with reagents CK19 [36]. Conversely, positivity for CK7 and CK20 take much lower proportion owing to that both of them indicate HCC diagnosis.

In terms of treatment, there are not any currently available data in the literature specifically pertaining to IHA due to its extreme rarity. Generally, the disease is treated using similar strategies as those used to treat CA. Radical surgery with simultaneous liver metastasis resection followed by systemic chemotherapy is considered the optimal treatment [45]. However, in comparison with CA, IHA presents a more challenging radical resection due to its higher frequency of lymph node and distant metastasis. Moreover, even with radical surgery and subsequent chemotherapy, IHA exhibited an astonishingly worse prognosis compared with CA. Several cases reported that patients usually died within the first 12 months; however, the exact data regarding the survival are quite limited. In this study, we collected the survival data of 36 cases of IHA, and determined the 1-/2-/3-year DSS were 30.6%, 22.9% and 7.6%, respectively, which indicated exceedingly worse prognosis compared with the 1-/2-/3-year DSS of CA (84.7%, 76.6% and 70.4%, respectively). Furthermore, in our study, we found that only pTNM stage was an independent risk factor for PFS and DSS. Surprisingly, the PFS and DSS of IHA had no relationship with chemotherapy, which may indicate that IHA is not sensitive to the typical chemotherapy agents used for intestinal cancers, sharing this behavior with the primary liver tumor counterpart.

For the 21 patients suffering distant metastasis in this study, the most common site was the liver, followed by the lung, which was similar to HAS. Strikingly, all of those patients with distant metastasis at the time of the initial diagnosis died within the first year. In another study of HAS performed by our same research group, we found that portal vein thrombosis and adjuvant therapy were independent risk factors for PFS and that complete resection, pTNM stage and adjuvant therapy were prognostic risk factors for DSS. In order to perform an unbiased comparison of the prognosis, patients were matched using propensity score matching with a one-to-one scheme to minimize intergroup differences regarding those baseline characteristics. The survival analysis showed that the PFS and DSS of IHA were significantly lower than HAS.

There were two limitations in the present study. First, this was a retrospective analysis, with the extent of data being relatively limited. Second, the sample size of patients with IHA was not large enough, which may have resulted in some statistical bias.

## CONCLUSIONS

IHA is a rare malignancy that most frequently occurs in the colon. The majority of cases presented with hematochezia and showed elevated serum AFP. IHA patients with IBD were common. Early pTNM stage predicted a more favorable survival. IHA differed exceedingly from CA, but was similar to that of HAS with respect to the clinicopathological characteristics. The prognosis of IHA was significantly worse than that of CA and HAS.

## PATIENTS AND METHODS

We retrospectively analyzed the clinical data of patients with IHA from our center, diagnosed between January 2010 and December 2016, as well as from the literature via the three largest medical databases (CBM disc, HowNet and Wanfang) in China, PubMed, Web of Science, Embase and the Cochrane Library in English, before February 2017. Cases that accompanied other intestinal malignancies and died during the postoperative period were excluded from our study. This study was approved by the ethics committee of Union Hospital, Tongji Medical College, Huazhong University of Science and Technology, in accordance with the 1964 Helsinki Declaration and its later amendments or comparable ethical standards, with written informed consent being obtained from all the patients from our institution.

Data including age, sex, initial presentation, tumor location, accompanying IBD, elevated of serum biomarkers, biopsy, tumor size, portal vein thrombosis, distant metastasis, surgical resection, differentiation, depth, lymph node metastasis, vascular invasion, pTNM stage, immunohistochemistry (IHC), neo-/adjuvant therapy, tumor progression, and survival data were recorded from our hospital medical records or extracted from published reports and studies. The pTNM tumor stage was reclassified according to the 7th edition of the American Joint Committee on Cancer (AJCC) [46].

The assessment of clinicopathological data was conducted with SPSS version 23.0 for Windows (IBM Corp., Armonk, NY, USA). Intergroup comparisons were analyzed using the chi-square test or Fisher's exact test, as appropriate. Continuous variables were expressed as the mean ± standard deviation (SD). The Kaplan-Meier method was used to evaluate both the progression-free survival (PFS) and disease-specific survival (DSS), with the log-rank test being used to examine the differences between survival curves. Significant predictors for survival, as identified by the univariate analysis were included in the multivariate analysis. PFS was defined from the date of treatment to the date of disease progression. DSS was defined from the date of surgery to the date of cancer-related death. A *P* value < 0.05 (two-tailed) was considered statistically significant.

## Abbreviations

AFP: alpha-fetoprotein
AJCC: American Joint Committee on Cancer
CA: colorectal adenocarcinoma
DSS: disease-specific survival
HAC: hepatoid adenocarcinoma
HAS: hepatoid adenocarcinoma of the stomach
IBD: inflammatory bowel disease
IHA: intestinal hepatoid adenocarcinoma
PFS: progression-free survival
SEER: Surveillance, Epidemiology, and End Results
